# Calculation of health expectancies with administrative data for North Rhine-Westphalia, a Federal State of Germany, 1999–2005

**DOI:** 10.1186/1478-7954-7-4

**Published:** 2009-03-19

**Authors:** Paulo Pinheiro, Alexander Krämer

**Affiliations:** 1 Department of Public Health Medicine, School of Public Health, University of Bielefeld, Universitätsstr. 25, 33615 Bielefeld, Germany

## Abstract

**Objectives:**

The main objectives of this study were to prove the feasibility of health expectancy analyses with regional administrative health statistics and to explore the utility of the calculated health expectancies in describing the health state of the population living in North Rhine-Westphalia, a Federal State of Germany.

**Materials and methods:**

Administrative population and mortality data as well as health data on disability and long-term care provided by public services were used to calculate: a) the life expectancy and b) the health expectancies Severe-Disability-Free Life Expectancy (SDFLE) and Long-Term-Care-Free Life Expectancy (LTCFLE) from 1999 to 2005. Calculations were done using the Sullivan method.

**Results:**

SDFLE at birth was 69.9 years (males 66.2 and females 73.2 years) in 1999 and it increased to 71.7 years (males 68.6 and females 74.7 years) in 2005. The proportion of the SDFLE on the total life expectancy at birth was 89.8% (males 88.6 and females 90.8%) in 1999 and 90.7% (males 89.8 and females 91.4%) in 2005.

LTCFLE at birth was 75.3 years (males 73.1 and females 77.5 years) in 1999 and it increased to 76.6 years (males 74.7 and females 78.6 years) in 2005. The proportion of the LTCFLE on the total life expectancy at birth was 96.8% (males 97.8 and females 96.1%) in 1999 and 96.8% (males 97.8 and females 96.2%) in 2005.

**Discussion and conclusion:**

Both health expectancies indicate an improvement in the quantity as well as in the quality of healthy life for the population living in North Rhine Westphalia and therefore suggest a compression of morbidity from 1999 to 2005. The findings however have several limitations in their sensitivity, since we applied dichotomous valuations to the health states. In addition, the results are restricted to comparisons over time because the morbidity concepts do not allow for comparisons with populations other than the German one. Refined calculations with other summary measures of population health and with health data on other morbidity concepts are therefore reasonable.

## Background

Mortality data have traditionally been an important source of information for describing the overall health of a population and for identifying health problems for a population. Over the last century, the health of populations living in economically developed countries has changed substantially. Life expectancies at birth have increased impressively as a result of declining overall death rates, and age-at-death and cause-of-death patterns have significantly altered with a shift of mortality to older ages and the rise of non-communicable diseases [1,2]. Hence, today more people live longer with serious illness and disability. Consequently, much public health attention has moved towards the quality of life-years gained, i.e. to the morbidity of a population [3,4], and merely looking at health indicators based on a population's mortality (e.g. life expectancy, infant death rates) is now no longer considered to be adequate for quantifying the overall health of a population [5,6].

In the past two decades, considerable international effort has therefore been put into the development, calculation and use of summary measures of population health [7,8]. Summary measures of population health (SMPH) are measures that combine information on mortality and non-fatal health outcomes to represent the health of a particular population as a single number [9]. The SMPH family can broadly be classified into two major classes: Health expectancies (e.g. healthy life expectancy) and health gaps (e.g. disability-adjusted life years). A health expectancy is defined as the average number of years that an individual is expected to be healthy at a certain age if current mortality and health status trends continue. It summarizes the total life expectancy into equivalent years of full health by taking into account years lived in less-than-full health states [10]. Since a health expectancy is the combination of a life expectancy with a health concept, there are theoretically as many health expectancy measures as health concepts. In contrast to a health expectancy, a health gap strives to estimate losses of health in populations by quantifying the difference between the current health status of a population and some stated, arbitrarily defined norm or goal for population health [11]. One such metric, the disability-adjusted life year (DALY), was developed by the World Bank and the World Health Organization (WHO) as part of the Global Burden of Disease Project [12] and has become some form of standard measure in burden of disease and injury studies [13-15].

Among the health expectancy indicators, particular importance has been attached to the measurement unit "healthy life years" (HLYs), since it was integrated into the core set of the European Union's (EU) structural indicators [16]. HLYs are calculated following the Sullivan method and are based on mortality data and on the age-specific prevalence of self-perceived disability in a population [17]. The Sullivan method offers several advantages over other SMPH methods: Its health expectancy indicator is relatively simple to calculate, readily interpretable, and independent of the size and age structure of the population [18].

Although SMPH have become a major subject in public health research and are routinely used in national and international public health policy-making and monitoring processes, there is still little awareness and use of these measures in Germany, by both public health academia, and national and regional health authorities. This report provides the use of the Sullivan method for calculating health expectancy indicators at a regional level in North Rhine-Westphalia, a Federal State of Germany. The main objectives of this study were twofold: First, to prove the feasibility of health expectancy analyses with regional health statistics. Second, to explore the utility of the calculated health expectancies in describing the health state of the population residing in North Rhine-Westphalia.

## Materials and methods

### Population

North Rhine-Westphalia (*Nordrhein-Westfalen*) is situated in the Western part of Germany and, with more than 18 million inhabitants, it is the most populous German federal state. It shares borders with Belgium and the Netherlands. Within Germany, it shares borders with the federal states of Lower Saxony to the North and Northeast, Rhineland-Palatinate to the Southwest and Hesse to the Southeast.

North Rhine-Westphalia was established as a federal state by the British military administration in 1946 when Germany was reorganised after World War II. Initially, it consisted of Westphalia and the northern part of the Rhine Province, both provinces formerly part of Prussia. In 1947, the former state of Lippe was merged with North Rhine-Westphalia, leading to the present administrative boundaries.

### Data

We used administrative data on population, mortality, and morbidity in North Rhine-Westphalia provided by the following public services: North Rhine-Westphalia Office for Data Processing and Statistics *(Landesamt für Datenverarbeitung und Statistik NRW*; ) and the North Rhine-Westphalia Institute of Public Health (*Landesinstitut für den öffentlichen Gesundheitsdienst NRW*; ).

The following data record was created in order to carry out calculations for the years 1999, 2001, 2003, and 2005:

• Population by sex and age (one-year age groups, 90 years and over age group) on 31st of December 1998, 1999, 2000, 2001, 2002, 2003, 2004, 2005

• Deaths by sex and age (one-year age groups; 90 years and over age group) during the years 1999, 2001, 2003, 2005

• Live births by sex during the years 1999, 2001, 2003, 2005

• Severely disabled (Degree of disability more than 50 as defined below) by sex and age group (five-year age groups; 90 years and over age group) on 31st of December 1999, 2001, 2003, 2005

• Nursing cases (Care level 1–3) by sex and age group (five-year age groups; 90 years and over age group) during the years 1999, 2001, 2003, 2005

### Health concepts, health states less than full health

#### Disability and severe disability

The concept of disability which is assessed by the German administrative statistics is based on definitions provided by German law. As defined in the German Social Code Volume IX (*Sozialgesetzbuch IX*), persons are disabled if their physical functions, mental capacities or psychological health are highly likely to deviate from the condition which is typical for the respective age for more than six months and, thus, whose participation in the societal life is restricted [19]. They are threatened by disability if this restriction is expected to occur. The impact of impairment on the physical, mental, psychological and social functional capacities is expressed in terms of the measure "Degree of Disability" (*Grad der Behinderung*) which is graded in tens from 20 to 100. Severely disabled persons are, according to the German Social Code, those persons whose degree of disability is at least 50 and who have either a legal residence or a legal occupation in Germany [19]. The degree of disability is determined by medical experts according to a standardised set of diagnostic criteria, which are defined in a national guideline [20]. The guideline provides the medical expert with a detailed description of a wide range of impairments that possibly affect the various organ systems. Also, the guideline informs about the degree of disability that is associated with a certain limitation and, thus, enables the expert to quantify the disability based on the patient history and on medical findings.

#### Need for long-term care

Need for long-term care (*Pflegebedürftigkeit*) can also be regarded as a less-than-full health status for which administrative data provided by public services are available in Germany. As defined in the German Social Code Volume XI (*Sozialgesetzbuch XI*), persons need long-term care if they are expected to depend on extensive help for at least six months in performing their common and periodic activities of daily life due to a physical, mental or psychological illness or disability [21]. To decide whether or not a person needs long-term care is the responsibility of the long-term care insurance funds. These funds have the severity of care needs assessed and verified by the Medical Review Board of the Statutory Health Insurance Funds (*Medizinischer Dienst der Krankenkassen*), which consists primarily of doctors and nurses [22]. The health status of a person is evaluated by these experts based on guidelines and it is then assigned to one of three care levels: extensive need (level I), severe need (level II), highly severe need (level III) for long-term care [23]. In our study, we used the data on the prevalence of people in the three care levels. Since long-term care is assessed and collected independently from disability, one has to keep in mind that some prevalence of people in long-term care was added to the prevalence of severe disability.

### Calculation of life and health expectancies

The collected data record was used to create the health expectancy indicators Severe-Disability-Free Life Expectancy (SDFLE) and Long-Term-Care-Free Life Expectancy (LTCFLE). Calculations were carried out using the Sullivan method which can be used to calculate health expectancies when information on the age-specific prevalence of the population in healthy and unhealthy states is available. We calculated total and healthy life expectancies using a calculation guide provided by the European Health Expectancy Monitoring Unit (EHEMU) [24]. In brief, the Sullivan method first requires the build-up of a period life table and hence the calculation of age and sex specific life expectancy values. These are derived from the calculation of the person years lived at a given age by a future cohort, assuming that the observed age-specific mortality rates apply. As a second step, the age and sex specific prevalence of a given health state is used to divide the corresponding person years lived into those lived with and without the given health state. Thus, the health expectancy is expressed in terms of the life expectancy that is free of a given health problem.

We calculated SDFLE and LTCFLE using unabridged life tables. As we did not have prevalence data in single years of age, we matched up the prevalence data to our life table data by assuming that each single year had the same prevalence as that age group. Mid year population was estimated by using the arithmetic mean of the population on the 31st of December of the year of reference and the population on the 31st of December of the preceding year. Information on the number of births in the year of reference was additionally required in order to calculate the probability of death between birth and age one, according to the EHEMU guidance [24].

## Results

### Life expectancy

**Figure 1:**
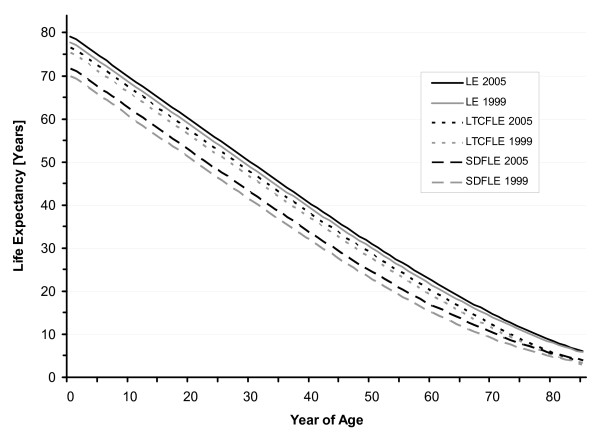
**Total (LE), Severe-Disability-Free (SDFLE), and Long-Term-Care-Free Life Expectancy (LTCFLE) in North Rhine-Westphalia, 1999 and 2005**.

**Figure 2:**
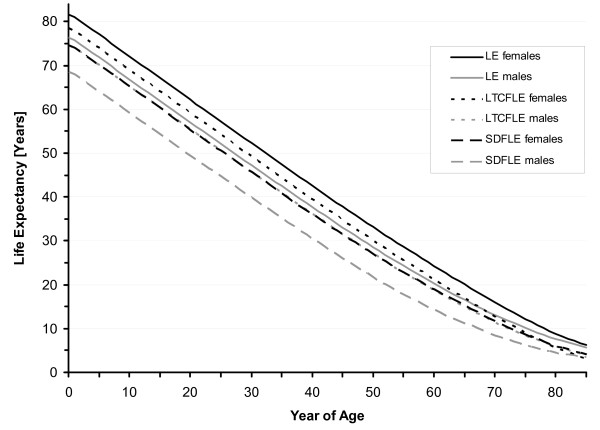
**Sex-specific total (LE), Severe-Disability-Free (SDFLE), and Long-Term-Care-Free Life Expectancy (LTCFLE) in North Rhine-Westphalia, 2005**.

In North Rhine Westphalia, life expectancy at birth was 77.8 years in 1999 and it increased to 79.1 years in 2005 (78.4 years in both 2001 and 2003). For males, life expectancy at birth was estimated to be 74.7 years in 1999 and 76.4 years in 2005 (75.4 years in 2001 and 75.6 years in 2003). The female life expectancy at birth was 80.7 years in 1999 and 81.7 years in 2005 (81.1 years in both 2001 and 2003) (Table 1, Figure 1 and Figure 2). From 1999 to 2005, life expectancy at birth increased by 1.3 years (1.7%). While the male life expectancy at birth increased by 1.7 years (2.3%) between 1999 and 2005, there was an increase of 1.0 years (1.2%) in the female life expectancy.

**Table 1:** Total (LE), Severe-Disability-Free (SDFLE), and Long-Term-Care-Free Life Expectancy (LTCFLE) at various years of age in North Rhine-Westphalia, 1999 and 2005

		**LE [years]**		**SDFLE [years]**		**LTCFLE [years]**	
		
		*1999*	*2005*	*1999*	*2005*	*1999*	*2005*
Total	At birth	77.8	79.1	69.9	71.7	75.3	76.6
	
	At 15 years of age	63.4	64.6	55.5	57.3	60.9	62.2
	
	At 65 years of age	17.5	18.5	11.8	13.4	15.0	16.0
	
	At 85 years of age	5.8	6.1	3.4	4.0	3.0	3.3

							

Males	At birth	74.7	76.4	66.2	68.6	73.1	74.7
	
	At 15 years of age	60.3	61.9	51.9	54.3	58.8	60.3
	
	At 65 years of age	15.3	16.6	9.1	11.2	13.7	15.0
	
	At 85 years of age	5.1	5.7	2.6	3.5	3.3	4.0

							

Females	At birth	80.7	81.7	73.2	74.7	77.5	78.6
	
	At 15 years of age	66.1	67.2	58.8	60.3	63.0	64.1
	
	At 65 years of age	19.2	20.0	13.8	15.2	16.0	16.9
	
	At 85 years of age	6.0	6.2	3.6	4.2	2.8	3.1

### Severe-Disability-Free Life Expectancy (SDFLE)

**Figure 3:**
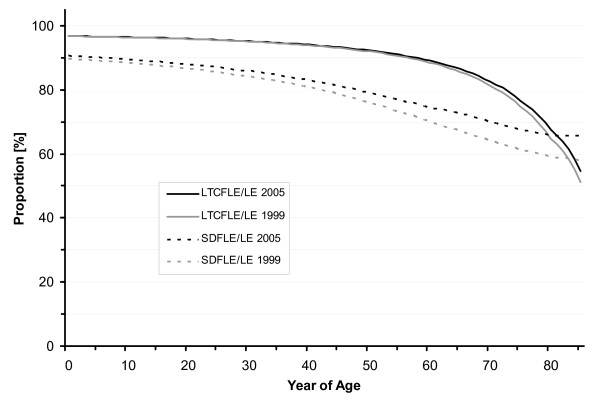
**Proportion of Severe-Disability-Free (SDFLE) and Long-Term-Care-Free Life Expectancy (LTCFLE) on total Life Expectancy (LE) in North Rhine-Westphalia, 1999 and 2005**.

**Figure 4:**
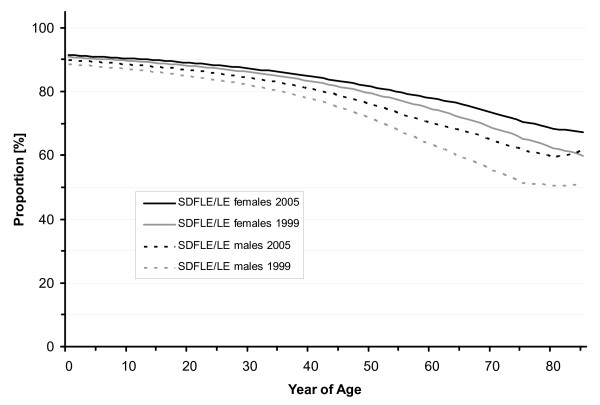
**Sex-specific proportion of Severe-Disability-Free (SDFLE) on total Life Expectancy (LE) in North Rhine-Westphalia, 1999 and 2005**.

**Figure 5:**
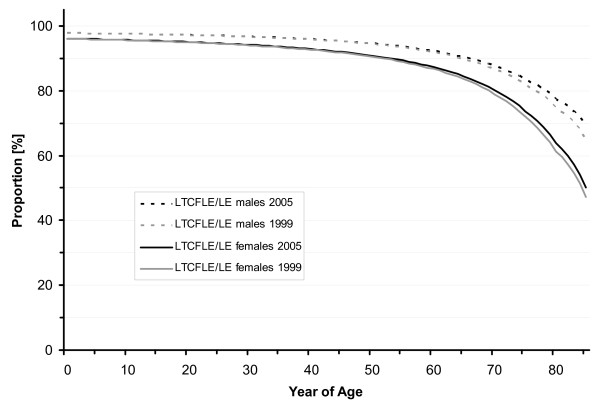
**Sex-specific proportion of Long-Term-Care-Free Life Expectancy (LTCFLE) on total Life Expectancy (LE) in North Rhine-Westphalia, 1999 and 2005**.

SDFLE at birth was 69.9 years (males 66.2 and females 73.2 years) in 1999 and it increased to 71.7 years (males 68.6 and females 74.7 years) in 2005. In 2001 and 2003, SDFLE at birth was estimated to be 70.5 (males 67.0 and females 73.7 years) and 71.2 years (males 67.9 and females 74.3 years), respectively (Table 1, Figure 1 and Figure 2). From 1999 to 2005, the SDFLE at birth increased by 1.8 years (2.6%). The male SDFLE at birth increased by 2.4 years (3.6%), and the female SDFLE at birth – by 1.5 years (2.0%). The proportion of the SDFLE on the total life expectancy at birth was 89.8% (males 88.6 and females 90.8%) in 1999 and 90.7% (males 89.8 and females 91.4%) in 2005 (Table 2, Figure 3, Figure 4 and Figure 5).

**Table 2:** Proportion of Severe-Disability-Free (SDFLE) and Long-Term-Care-Free Life Expectancy (LTCFLE) on total Life Expectancy (LE) at various years of age in North Rhine-Westphalia, 1999 and 2005

	**SDFLE/LE in %**					
	
	**Total**		**Males**		**Females**	
	
	*1999*	*2005*	*1999*	*2005*	*1999*	*2005*
At birth	89.8	90.7	88.6	89.8	90.8	91.4

At 15 years of age	87.6	88.8	86.0	87.7	88.9	89.7

At 65 years of age	67.1	72.7	59.2	67.8	71.8	76.0

At 85 years of age	57.9	65.7	51.1	61.6	59.9	67.2
	
						
	
	**LTCFLE/LE in %**					
	
	**Total**		**Males**		**Females**	
	
	*1999*	*2005*	*1999*	*2005*	*1999*	*2005*

At birth	96.8	96.8	97.8	97.8	96.1	96.2

At 15 years of age	96.1	96.2	97.4	97.4	95.3	95.4

At 65 years of age	85.6	86.4	89.7	90.4	83.6	84.4

At 85 years of age	51.5	54.6	64.9	69.4	47.3	50.0

### Long-Term-Care-Free Life Expectancy (LTCFLE)

LTCFLE at birth was 75.3 years (males 73.1 and females 77.5 years) in 1999 and it increased to 76.6 years (males 74.7 and females 78.6 years) in 2005. In 2001 and 2003, LTCFLE at birth was 75.8 (males 73.8 and females 77.9 years) and 75.9 years (males 73.9 and females 78.0 years), respectively (Table 1, Figure 1 and Figure 2). Between 1999 and 2005, LTCFLE at birth increased by 1.3 years (1.7%). For males, LTCFLE at birth increased by 1.6 years (2.2%), and for females it increased by 1.1 years (1.4%). The proportion of the LTCFLE on the total life expectancy at birth was 96.8% (males 97.8 and females 96.1%) in 1999 and 96.8% (males 97.8% and females 96.2%) in 2005 (Table 2, Figure 3, Figure 4 and Figure 5).

## Discussion

Health expectancies are increasingly being recognized as useful indicators for describing a population's health, as they integrate information on the morbidity and mortality of a population into one single measure [7]. In doing so, health expectancies allow for estimates to be more comprehensive than those that have traditionally been made with mortality-based health indicators, such as life expectancy. The findings of our study contribute to a refined view on the health status of the population living in North Rhine-Westphalia, a Federal State of Germany, and they demonstrate that estimates of health expectancies based on administrative data provided by public services are feasible at a regional level in Germany.

Our results firstly show that the total as well as two different "healthy" life expectancies increased in North Rhine-Westphalia from 1999 to 2005. While the total life expectancy at birth increased by 1.3 years to 79.1 years in 2005, there was an increase of 1.8 years to 71.7 years in the SDFLE at birth and of 1.3 years to 76.6 years in the LTCFLE at birth (see Figure 1). Our results also suggest that the quality of life, when defined as the proportion of time that one is expected to live free of severe disability or long-time care during life, has improved between 1999 and 2005. While the proportion of the LTCFLE on the total life expectancy showed minor improvements, the increase in the proportion of the SDFLE on the life expectancy was more pronounced (see Figure 3). Thus, our findings would add to the compression-of-morbidity hypothesis if morbidity was reflected by the health concepts used in our study.

As expected, the absolute expectancy values were lower for the male population than those for females. In 2005, the sex-specific differences were 5.3 years in life expectancy at birth, and 6.1 and 3.9 years in SDFLE and LTCFLE at birth, respectively. On the other hand, the results from our study also indicate that the health status of the male population has improved more significantly than the health status of the female population between 1999 and 2005. The gains in life and health expectancies were generally higher for men in terms of absolute life years gained as well as in terms of the relative growth since 1999. SDFLE and LTCFLE showed different characteristics for males and females. SDFLE was notably low in men at all years of age. Accordingly, the percentage of SDFLE on total life expectancy was considerably lower for men as compared to women. Regarding LTCFLE, the absolute values were also lower for men than for women, but the proportion of LTCFLE on total life expectancy was higher for men at all years of age.

Improvements in the life and health expectancies from 1999 to 2005 benefited especially the population at advanced years of age. The percentage increase of SDFLE and LTCFLE at 65 and 85 years of age was significantly higher than the relative growth of SDFLE and LTCFLE at birth and at 15 years of age. The decline in the proportion of the LTCFLE on the total life expectancy was obvious in the older and oldest population (see Figure 5). whereas the decrease in the SDFLE values also affected the middle age groups. On the other hand, our findings on the proportion of the SDFLE on the total life expectancy indicate that especially the older and oldest male population living in the year 2005 experienced improvements in their quality of life (see Figure 4). One possible explanation for this trend could be the fact that, among the men who died between 1999 and 2005 at 80 and more years of age, there was a high proportion of severely disabled men from World War II.

The interpretation of our results, however, encounters several limitations. One is the question if the improvements in the health status, as indicated by our results, are improvements that really benefited the population living in North Rhine-Westphalia. An affirmative answer implicitly includes the assumption that the valuation of the health states less than full health, i.e. severe disability and long-term care, did not change over the analysed period of time. For both ill-health states, valuation was conducted by medical experts to see if a person qualifies for statutory insurance benefits.

In addition, the present findings do not allow for information on the overall health of a population and are restricted to the dimensions of health assessed. In this context, the terms health expectancy or healthy life expectancy might be misleading because they suggest results that allow for a generalised description of health. When using life expectancies free of a certain ill-health state to measure population health as we did, the interpretation of results has to keep in mind that the apparently healthy life expectancy does not consider ill-health states other than the analysed ones.

Furthermore, the comparability of our results is restricted to the health status of our population over time or to different populations within Germany at the same time. The health expectancies from our study are not comparable to similar health expectancies, such as the EU indicator HLY. Although disability data and the Sullivan method were used for calculating the SDFLE in our study and are also the basis for HLY calculations, we used expert-based valuations of disability, while HLYs are based on self-perceived disability assessed by health surveys.

The data record did not allow for stratification other than sex and age because further information on the population of North Rhine-Westphalia (e.g. the socioeconomic status linked to health-related data) was not available. To address this limitation, there is the possibility to calculate LE, SDFLE and LTCFLE for sub-regions of North Rhine-Westphalia that have recently been classified based on cluster analyses and that differ in their socio-demographic profiles [25].

In the present study, the Sullivan method was used to compute the health expectancies SDFLE and LTCFLE. One key characteristic of our study was the fact that we applied dichotomous valuations to the states of ill health. Information was therefore restricted to the presence or absence of ill health. Basically, dichotomous valuations make the health expectancy measure extremely dependent on the variation in the threshold that is usually arbitrarily defined. Up to the threshold the valuation is zero and equivalent to the valuation of death or no health, and beyond that threshold the valuation is one and equivalent to full health [26]. When using administrative data for assessing time trends in health expectancies, one has to keep in mind that changes in the eligibility criteria for the scheme, whether legislated or informal, can affect trends in the prevalence of people living in a state of ill health.

Furthermore, the use of dichotomous valuations automatically resulted in a limitation of the sensitivity of our results. We did not apply gradual valuations to the states of ill health for calculating our health expectancies, although the data record on disability and long-term care provided some. Hence, sensitivity can be improved when making refined calculations by applying weights to the several health states. This would then allow for calculating life expectancies adjusted on a state of ill health, such as the indicator disability adjusted life expectancy DALE.

In summary, we could demonstrate that the calculation of health expectancies with administrative data from public services was feasible at a regional level. Moreover, the results, albeit restricted by a range of limitations, added some new insights into the health status of the population living in North Rhine-Westphalia. We thus conclude that our future work on health expectancies should target, firstly, the use of refined methods that allow the integration of gradually assessed health data and, secondly, the extension of the range of health expectancies including the use of standardised measures to make comparisons with populations other than the German one possible.

## Competing interests

The authors declare that they have no competing interests.

## Authors' contributions

PP contributed to the design of the study, performed the data acquisition, analysis, and interpretation and drafted the first version of the manuscript. AK contributed to the interpretation of findings and the revision of the manuscript. Both authors read and approved the final manuscript.

## References

[B1] Omran AR (1971). The epidemiologic transition. A theory of the epidemiology of population change. Milbank Mem Fund Q.

[B2] Olshansky SJ, Ault AB (1986). The fourth stage of the epidemiologic transition: the age of delayed degenerative diseases. Milbank Q.

[B3] Fries JF (1980). Aging, natural death, and the compression of morbidity. N Engl J Med.

[B4] Robine JM, Michel JP (2004). Looking forward to a general theory on population aging. J Gerontol A Biol Sci Med Sci.

[B5] Mathers CD, Salomon JA, Murray CJ (2003). Infant mortality is not an adequate summary measure of population health. J Epidemiol Community Health.

[B6] Murray CJ (1988). The infant mortality rate, life expectancy at birth, and a linear index of mortality as measures of general health status. Int J Epidemiol.

[B7] Robine JM, Jagger C, Mathers CD, Crimmins EM, Suzman RM, (Eds) (2003). Determining Health Expectancies.

[B8] Murray CJL, Salomon JA, Mathers CD, Lopez AD, (Eds) (2002). Summary Measures of Population Health; Concepts, Ethics, Measurement and Applications.

[B9] Field MJ, Gold MR, (Eds) (1998). Summarizing Population Health – Directions for the Development and Application of Population Metrics.

[B10] Mathers CD, Murray CJL, Salomon JA, Mathers CD, Lopez AD (2002). Health expectancies: an overview and critical appraisal. Summary Measures of Population Health; Concepts, Ethics, Measurement and Applications.

[B11] Murray CJL, Mathers CD, Salomon JA, Lopez AD, Murray CJL, Salomon JA, Mathers CD, Lopez AD (2002). Health gaps: an overview and critical appraisal. Summary Measures of Population Health; Concepts, Ethics, Measurement and Applications.

[B12] Murray CJL, Lopez AD, (Eds) (1996). The Global Burden of Disease: a comprehensive assessment of mortality and disability from diseases, injuries, and risk factors in 1990 and projected to 2020.

[B13] McKenna MT, Michaud CM, Murray CJ, Marks JS (2005). Assessing the burden of disease in the United States using disability-adjusted life years. Am J Prev Med.

[B14] Bradshaw D, Groenewald P, Laubscher R, Nannan N, Nojilana B, Norman R, Pieterse D, Schneider M, Bourne DE, Timaeus IM, Dorrington R, Johnson L (2003). Initial burden of disease estimates for South Africa, 2000. S Afr Med J.

[B15] Stevens G, Dias RH, Thomas KJ, Rivera JA, Carvalho N, Barquera S, Hill K, Ezzati M (2008). Characterizing the epidemiological transition in Mexico: national and subnational burden of diseases, injuries, and risk factors. PLoS Med.

[B16] Healthy Life Years. http://ec.europa.eu/health/ph_information/indicators/lifeyears_en.htm.

[B17] Sullivan DF (1971). A single index of mortality and morbidity. HSMHA Health Rep.

[B18] Barendregt JJ, Robine JM, Jagger C, Mathers CD, Crimmins EM, Suzman RM (2003). Disability-adjusted Life Years (DALYs) and Disability-adjusted Life Expectancy (DALE). Determining Health Expectancies.

[B19] (2001). Neuntes Buch Sozialgesetzbuch – Rehabilitation und Teilhabe behinderter Menschen – (Artikel 1 des Gesetzes vom 19 Juni BGBl I S 1046), § 2 Behinderung.

[B20] Bundesministerium für Gesundheit und Soziale Sicherung (2008). Anhaltspunkte für die ärztliche Gutachtertätigkeit im sozialen Entschädigungsrecht und nach dem Schwerbehindertenrecht.

[B21] Sozialgesetzbuch (SGB) – Elftes Buch (XI) – Soziale Pflegeversicherung (Artikel 1 des Gesetzes vom 26 Mai 1994, BGBl I S 1014), § 14 Begriff der Pflegebedürftigkeit.

[B22] Sozialgesetzbuch (SGB) – Elftes Buch (XI) – Soziale Pflegeversicherung (Artikel 1 des Gesetzes vom 26 Mai 1994, BGBl I S 1014), § 18 Verfahren zur Feststellung der Pflegebedürftigkeit.

[B23] Sozialgesetzbuch (SGB) – Elftes Buch (XI) – Soziale Pflegeversicherung (Artikel 1 des Gesetzes vom 26 Mai 1994, BGBl I S 1014), § 15 Stufen der Pflegebedürftigkeit.

[B24] Jagger C, Cox B, Le Roy S EHEMU: Health Expectancy Calculation by the Sullivan method: A Practical Guide EHEMU Technical Report September 2006.

[B25] Strohmeier KP, Schultz A, Bardehle D, Annuss R, Lenz A (2007). [Health indicator-based cluster analysis of districts and urban districts in North Rhine-Westphalia]. Gesundheitswesen.

[B26] Murray CJ, Salomon JA, Mathers C (2000). A critical examination of summary measures of population health. Bull World Health Organ.

